# Current trends on prevalence, risk factors and prevention of oral cancer

**DOI:** 10.3389/froh.2024.1505833

**Published:** 2024-11-13

**Authors:** Ricardo D. Coletta, W. Andrew Yeudall, Tuula Salo

**Affiliations:** ^1^ Department of Oral Diagnosis and Graduate Program in Oral Biology, School of Dentistry, University of Campinas, Piracicaba, Brazil; ^2^ Department of Oral Biology & Diagnostic Sciences, The Dental College of Georgia at Augusta University, and Georgia Cancer Center, Augusta, GA, United States; ^3^ Research Unit of Population Health, Department of Oral Pathology, University of Oulu and Oulu University Hospital, Oulu, Finland; ^4^ Medical Research Center, Oulu University Hospital, Oulu, Finland; ^5^ Department of Oral and Maxillofacial Diseases, and Translational Immunology Program (TRIMM), University of Helsinki, Helsinki, Finland; ^6^ iCAN Digital Precision Cancer Medicine Flagship, University of Helsinki, Helsinki, Finland

**Keywords:** oral cancer, prevalence, risk factor, prevention, challenges

## 1 Introduction

Oral squamous cell carcinoma (OSCC), belonging to the large and highly heterogeneous group of cancers in head and neck, corresponds to the tumors arising in the mucosal epithelium of the lips, buccal mucosa, hard palate, oral tongue (anterior 2/3 of the tongue), floor of mouth, gingiva and retromolar trigone. The most common oral sites affected by OSCC are lateral border of the oral tongue and buccal mucosa, depending on risk factors, with the first being associated mainly with cigarette smoking and the second with chewing tobacco (1). Lip cancer is also highly prevalent in tropical countries (but not restricted to them) due to chronic exposure to solar radiation, but as it frequently shows a very good prognosis, studies traditionally separate it from intraoral lesions. Indeed, nowadays, we have enough evidence that tumors arising in the different subsites of the oral cavity show distinct and specific features and they should be studied separately (2).

OSCC holds the poorest prognosis amongst head and neck cancers, and low survival rates remain unchanged for decades (3). Tumors frequently present at an advanced stage, with patients displaying locoregional disease. Although the revolutionizing targeted therapy and immunotherapy bring new perspectives for OSCC treatment, surgery in combination with radiotherapy and chemotherapy remains the primary treatment modality, which can promote chronic and lifelong morbidities with important impacts in the quality of life of patients who survive OSCC. Diagnosis at an early stage (T1 or T2 with no or limited nodal disease) remains the best predictor for successful treatment.

## 2 Prevalence of OSCC

With global incidence exceeding 389.000 new cases annually, OSCC remains one of the most common tumors worldwide, and this scenario is projected to get worse, as the incidence is estimated to rise 65% by 2050, as estimated by the [(4) GLOBOCAN]. Prevalence fluctuates significantly depending on the population, with crude rates ranging from 1.1 in African populations to 9.9 in populations from Oceania. Moreover, the burden is distinct between different countries, with a clear association with human development index (HDI) and both incidence and mortality. Regarding incidence, though an increase is expected for all HDI tiers, the increase in 2050 is expected to be approximately 147.8% in low HDI countries, 94.2% in medium HDI countries, 67.3% in high HDI countries, and only 34.3% in very high HDI countries. These trends in conjunction with global disparities in therapeutic options call urgently for strategies to mitigate the growing perspective of deaths due to oral cancers in low and very low HDI countries in the coming decades.

## 3 Risk factors

OSCC shows a predominance in men (approximately 2.5:1), and in both male and female cases the highest incidence is reported after the fifth decade of life. However, increasing incidence in women, younger adults and people not exposed to traditional risk factors, has been consistently reported in the last two decades [for more details see (3)]. Tobacco—any type ranging from cigarette to smokeless tobacco and betel quid chewing, with or without tobacco, as widely used in India, other Asia countries and in the Western Pacific islands—and alcohol consumption are the strongest risk factors driving oral carcinogenesis, and are considered the main criteria to define individuals at high-risk of developing oral cancer. Use of electronic cigarettes (e-cigarettes or also call vapes) became very popular in recent years, mainly among teenagers and young adults, and is currently the most commonly used tobacco product in this demographic in the United States (https://www.ncbi.nlm.nih.gov/books/NBK538684/), and in many other developed countries (5). Some e-cigarettes may lack nicotine, which comes from tobacco, but the chemical complexity of the liquids and aerosols may, nevertheless, contain hazardous components such as the solvents propylene glycol and glycerol, flavoring chemicals, color additives and synthetic sweeteners, among other ingredients (6). Although evidence regarding the impact of e-cigarettes on cancer, including oral cancers, is limited, a case of OSCC in a young adult with an extensive history of vaping was reported (7), and several studies have reported quite similar oral diseases associated with traditional tobacco in e-cigarette users, including xerostomia, oral dysbiosis, progression of periodontal diseases and negative impact on dental implant outcomes (8–11). To date, the limited evidence suggests that e-cigarettes are harmful and should not be considered risk-free for cancer development, and users should be warned about the oral risks by dentists. Only long-term follow-up in well-designed studies will really provide a clear picture of the impact of e-cigarettes on OSCC occurrence; in the meantime, education and continued surveillance of e-cigarette users are needed.

Other OSCC risk factors have been suggested, including chronic oral inflammatory conditions and human papillomavirus (HPV), but the current literature remains limited, variable in quality and often underpowered. Chronic conditions promoting an oral inflammatory response such as mechanical trauma/irritation or oral dysbiosis have been described as possibly implicated in the development of OSCC, and the rationale is that multiple genetic and epigenetic changes may be induced by oxidative stress and signaling molecules related to modulation of the innate and acquired immune response (12). In oral dysbiotic conditions, there are also pathogen-derived genotoxins and carcinogens that may trigger oncogenic events. However, the evidence to explicitly prove that chronic oral inflammatory conditions promote and/or drive progression of OSCCs is still limited, and further detailed studies are necessary to elucidate the existing connection between these and OSCC. Although the oncogenic potential of HPV is unquestionable, particularly in oropharyngeal squamous cell carcinoma (which is closely related to OSCC), HPV studies of OSCC have largely focused on HPV-DNA detection and genotyping, with very few exploring the transcriptional activity and triggered pathways that underpin its oncogenic potential. Such studies have clearly contributed to an overestimation of the real contribution of HPV to OSCC carcinogenesis. A study with 3.680 samples from 29 countries from Europe and the Americas identified transcriptionally active HPV, which was assessed by using either or both E6*I mRNA quantification or p16^INK4a^ immunohistochemical expression, in combination to HPV-DNA detection, in only 3.0%–4.4% of oral cavity cancers, mainly represented by OSCC (13). A similar trend was observed in a Belgian study, where the fraction of OSCC attributed to HPV was 3.0% (14), whereas in a Brazilian cohort, transcriptionally active HPV was not detected in any of 89 OSCC cases (15). In a Norwegian cohort of oral tongue cancers, no transcriptionally active HPV was detected, but p16 positivity with varying staining intensity and subcellular localization was detected in 42% of the cases (16). It is strongly recommended that studies exploring the participation of HPV on oral carcinogenesis should not be restricted to detection of HPV-DNA, but rather include analysis of transcriptional activity and regulation of related cellular pathways, such as those regulated by pRb and p53 (17). Only by adopting well-planned and standard protocols will we be able to determine the exact contribution of HPVs to OSCC occurrence.

## 4 Prevention of oral cancer

Both primary and secondary prevention are top priorities for oral cancer. Historically primary prevention has focused on tobacco cessation and discontinuing heavy alcohol consumption, whereas secondary prevention is concentrated on early diagnosis or, even better, on the diagnosis and management of oral potentially malignant disorders. In 2022, the article of the IACR working group set to revise oral cancer prevention strategies confirmed the benefits of quitting tobacco use and alcohol consumption on oral cancer risk, with decreased risk with increasing time since cessation (18). Although the evidence was limited, the IACR working group also highlighted the benefits of cessation in the use of areca nut products, with or without tobacco.

HPV vaccines were originally developed for prevention of cervical cancers, but their benefits can have broader scope, in prevention of other HPV-associated diseases, including anogenital cancers in men, oropharyngeal cancers and OSCC (19). The HPV vaccines are able to prevent HPV acquisition and, to date, three types of vaccines, ranging from protection of 2 subtypes to 9 subtypes HPV, are available and recommended for both females and males (20). The importance of HPV vaccination is unquestionable for preventive medicine, but the impact on oral cancer incidence, if it occurs, is only expected to become apparent in the next decades.

The diagnosis of OSCC at an early stage is still the most effective manner to reach cure, improve survival and reduce morbidity. In this context, oral cancer screening programs, as a strategy for early detection of oral cancer, have been developed. These programs include both general populations or individuals specifically at risk for developing an OSCC, which includes those with a long history of tobacco or alcohol consumption. Although the results showed effectiveness in diagnoses of cases at early stages in studies with both general and high-risk populations, studies with high-risk individuals showed better results (21). A recent systematic review focused on costs of oral cancer screening strategies revealed that the programs are cost-effective, especially when focused in individuals with a high-risk profile (22). Although the prevalence of the disease in a population should be taken into account when determining the usefulness and cost-effectivity of screening programs, even for countries with a very high occurrence of oral cancers, including India and Brazil, the effectivity of screening programs was superior when performed in the high-risk group for OSCC development (23, 24).

When defining individuals who are at high-risk for OSCC, besides smokers and heavy drinkers, patients with hereditary diseases including dyskeratosis congenita and Fanconi anemia, and with cancer syndromes such as Li-Fraumeni and Plummer-Vinson syndromes, should be considered. The primary mode for secondary prevention is visual clinical examination, but promising results on non-invasive or minimally invasive OSCC biomarkers in saliva, blood, buccal swabs and other body fluids of the patients (liquid biopsy) have been reported (25). Furthermore, artificial intelligence (AI) strategies, including clinical imaging modalities, smartphone-based technologies and histopathological images for diagnosis, have been used for screening and diagnosis of oral cancers (26). For more effective OSCC early detection programs, an accurate definition of high-risk individuals by integrating molecular knowledge and risk stratification profiles should be incorporated, and innovations that are becoming available should be integrated into the system, making the laborious and expensive process effective and more accessible for all countries.

Approximately 90% of OSCCs are attributable to smoking and consumption of alcohol, but it is unlikely that we can eliminate them from our society. Even so, we still have a portion of cases which are unrelated to them. The search for a universal and unequivocal strategy for oral cancer prevention continues, whereas early diagnosis remains as an important means of promoting patient survival, reducing mortality and morbidity. The integration of what is already known and what has yet to be uncovered will direct the future of the field.

## 5 Conclusions

Despite major advances and development of multiple diagnostic and therapeutic strategies, oral cancer still contributes to a large number of cancer cases and deaths around the world, and the future is alarming, as its incidence is expected to surge 65% by 2050. This trend is related to the continuous exposure to the traditional risk factors, but it may also be driven by the growing and aging of the world's population. In low and very low HDI countries, where the population has limited access to an ideal healthcare system, it is more likely that the expected incidence increase will underpin more significant negative impacts. Effective educational programs for preventing tobacco and alcohol use among youth are important, and early detection is still the best way to prevent mortality and morbidity, which can be achieved through national screening programs focused on high-risk subjects. Inclusion of minimally invasive biomarkers and new AI technologies hold promise for improving diagnosis in resource-limited settings.
